# Comparison of three different hpv self-sampling tools – a subanalysis of the prospective, randomized hannover self-collection study

**DOI:** 10.1007/s00404-026-08457-5

**Published:** 2026-06-25

**Authors:** Stephanie Hempel, Sophia Groß, Lena Steinkasserer, Leonie Theis, Yvonne Ziert, Armin Koch, Peter Hillemanns, Matthias Jentschke

**Affiliations:** 1 Department of Gynaecology and Obstetrics, Hannover Medical School, Carl-Neuberg-Straße 1, 30625 Hannover, Germany; 2 Department of Gynaecology and Obstetrics, University Medical Center Ulm, Prittwitzstraße 43, 89081 Ulm, Germany; 3 Department of Nephrology, Düsseldorf University Hospital, Moorenstr. 5, 40225 Düsseldorf, Germany; 4 Institute for Biometry, Hannover Medical School, Carl-Neuberg-Straße 1, 30625 Hannover, Germany; 5 Department of Gynaecology and Obstetrics, Klinikum Würzburg Mitte, Salvatorstr. 7, 97074 Würzburg, Germany

**Keywords:** Papillomaviridae, Cervical cancer screening, Female, Vaginal smear, Urine specimen collection, Cervix

## Abstract

**Objective:**

Cervical cancer and its precursor lesions are treatable if detected early; however, screening participation for high-risk human papillomavirus infections (hr-HPV) remains low. In 2021, Germany recorded 4,544 new cervical cancer cases and 2,071 related deaths. The HaSCo study evaluates the feasibility of HPV self-testing to improve screening participation. This sub-study evaluates performance and acceptability of three self-testing tools.

**Methods:**

This prospective, randomized sub-study examined Evalyn-Brush, FLOQSwabs, and first-void urine Colli-Pee among women aged 30—65 in Hannover, Germany. Addresses from the residents´ registration office were randomized into seven age groups and an 80/20 city–region distribution. A total of 19,995 women were assigned to opt-in (request a self-test) or opt-out (receive a test directly). Participation was requested from women without regular screening in the past two years.

**Results:**

1,860 samples were returned (9.3%). Colli-Pee (10.4%) and FLOQSwabs (10.1%) had similar return rates, while Evalyn showed significantly lower rates (7.4%). Screening frequency didn´t significantly affect return rates (p = 0.1825), although FLOQSwabs showed higher return rates among underscreened women. Invalid sample rates were low, highest for FLOQSwabs (1.67%). A total of 145 samples tested positive for hr-HPV (7.9%). Evalyn collected highest DNA content (p < 0.0001). Colli-Pee was most preferred, and 66.7% of participants favored self-testing.

**Conclusion:**

HPV self-tests were highly accepted and effective for collecting sufficient DNA material. Direct provision of user-friendly self-tests may support screening participation, particularly among underscreened women. All three devices performed strongly and appear suitable for integration into cervical cancer screening programs.

**Supplementary Information:**

The online version contains supplementary material available at 10.1007/s00404-026-08457-5.

## Take home message

**Table:** 

Self sampling using Evalyn Brush, FLOQSwabs, and Colli-Pee provides reliable HPV detection and high acceptability, offering a feasible tool to improve cervical cancer screening uptake.	

## Introduction

Cervical cancer (c.c.) is the fourth most common type of cancer and the fourth leading cause of cancer death among women, with an incidence rate of 18.8 per 100,000 worldwide. In 2021, Germany, recorded 4,544 new c.c. cases and 2,071 related deaths, an incidence of 7.7/100,000 [1, 2].

Primary cause for this cancer is a persistent high-risk human papillomavirus (hr-HPV) infection, with a transition period of 10–20 years. This offers an excellent opportunity for screening, early detection, and prevention [3]. There are over 15 genital HPV-types associated with c.c., but only 14 high-risk types are commonly detected in clinical HPV-screening assays [4]. HPV is categorized as high-risk (oncogenic) or low-risk (non-oncogenic) based on its association with c.c. and precursor lesions. HPV-16 and 18 are most oncogenic (70%). Only small percentages of HPV-infections are persistent, with majority resolving spontaneously within 12 to 36 months [5–8].

Preventive strategies (e.g. vaccination or screening) could prevent nearly 100% of HPV-related lesions [9]. Therefore, WHO advocates for eliminating c.c. (90—70—90 strategy). Low screening participation remains a key barrier. Two in three women aged 30–49 years have never been screened for c.c. worldwide [10]. Additionally, there is a significant disparity in screening rates (nine times higher in high-income countries (HICs)) [11]. Still, screening participation remains suboptimal in numerous HICs [12].

In Germany cervical cancer incidence decreased since implementing screening in 1971, however, it remains relatively high for a HIC [2, 13, 14]. Screening participation isn´t centrally registered in Germany, complicating identifying non-responders (women not participating in screening). A study in Lower Saxony (2015) investigated participation rates in c.c. screening, using health insurance (AOK Niedersachsen) data from 2006 until 2011. They identified yearly participation of 44.8% to 46.6% [15]. In comparison, a UK study (2012), using data from 2008 and 2009, demonstrated screening rates of 83.5% [16]. Need for screening, particularly among older women (> 65 years), is highlighted by a recent study in Germany (2023), where 47.4% of c.c. cases aged 60– 79 didn´t participate in screening ten years prior to diagnosis, associated with lower 5-year survival (76.7% versus 46.9%) [17].

Reasons for nonparticipation vary from avoidance of speculum examination (most common), lack of insurance, office hours, communication barriers, transportation problems, shame or pain, religious or cultural factors, male healthcare providers, fear of examination, history of sexual trauma, to low priority [16, 18, 19].

Self-testing for HPV-infections could drive WHO's ambitious strategy and reduce national, racial, and social disparities by lowering barriers to screening participation [3, 18].

Finding an optimal approach to integrating HPV self-testing into healthcare systems is a complex endeavor [9]. Currently, integration of HPV self-testing in screening programs is relatively low (as of 2021, only 17 of 139 countries) [11, 20].

Identifying appropriate tools for sample collection represents a significant challenge. Several devices and various settings have been assessed [21]. Results and techniques, however, differ [21–26]. All devices harvest exfoliated cells from the cervicovaginal canal for subsequent HPV-DNA detection [3]. Important factors to consider include type of device [25, 27], resuspension media [24], storage time and temperature before testing [22, 23].

In this prospective, randomized controlled sub-study, we compared three self-testing tools in the region of Hannover, Germany. Two dry self-sampling tools (Evalyn-Brush, FLOQSwabs), and one first-void urine collection tool (Colli-Pee). These were pre-validated in a clinical study in 2020. Revealing, all invasive cancer cases and over 90% of CIN 3 lesions tested positive for hr-HPV with all three self-collection devices [28]. In this sub-study we focused on frequency of invalid samples, DNA content, and user-friendliness.

## Material and methods

### HaSCo study:

Home-based HPV self-testing was evaluated in a prospective, randomized study design. Focus was on non-responders to regular screening programs. We compared performance and user-friendliness of three HPV self-testing tools. HaSCo study was registered at the German Clinical Trials Register (DRKS, registration number DRKS00019200) and approved by the institutional review board at Hannover Medical School (No. 8692_BO_S_2019).

Screening participation isn´t centrally registered in Germany. Therefore, addresses obtained from residents´ registration office were randomized into seven equally sized age groups (30—34, 35—39, 40—44, 45—49, 50—54, 55—59, > = 60) and an 80/20 city–region distribution. Then 19,995 women were randomly assigned to opt-in (request a self-test by mail, phone or online) or opt-out (receive a test, user manual and information letter directly). All participants received a questionnaire on personal characteristics, previous cancer screening participation, self-test satisfaction (suppl. material), written consent, and an enclosed prepaid envelope. Only women without a regular screening visit during the last two years were asked to participate. For this subanalysis, however, all returned samples (n = 1,844) were included, as the aim was to compare sample quality and user-friendliness of three self-sampling devices. Women with annual screening participation (Colli-Pee: 370; Evalyn: 282; FLOQSwabs: 356) were excluded only from the main HaSCo analysis.

Since HPV-positive women had to be notified, complete anonymization wasn´t feasible, but samples were pseudonymized before testing (via a specific number and barcode).

Supplementary material: a consort checklist and flowchart for allocation, inclusion, and analysis.

### Self-tests:

The selected self-tests were dry swabs, Evalyn-Brush (Rovers Medical Devices, KV Oss., Netherlands), FLOQSwab (Copan Italia, Italy), and first-void urine test, Colli-Pee (Novosanis, Wijnegem, Belgium).

Evalyn-Brush features flexible bristles to enhance absorption, cell collection and comfort. The handle incorporates two wings; maximizing insertion length, ensuring accurate sample collection and instilling user confidence. It´s inserted into the vagina, extended, and rotated, then retracted into the handle to prevent contamination during disposal or packaging, and sealed with a cap.

FLOQSwab has a tip flocked with nylon fibers arranged perpendicularly. These short, evenly distributed fiber strands create a thin, absorbent layer for effective collection, ensuring optimal sample absorption and release. It´s inserted into the vagina, rotated along its walls, and stored in a small plastic tube.

Colli-Pee FV-5000 collects the first 16.5 to 23 ml of initial urine stream. It consists of a collection canister (prefilled with 7 ml of UCM preservative) and a float system redirecting remaining urine past the tube, eliminating the need to interrupt urination. The prefilled preservative helps maintain stability of sample (DNA) at room temperature for up to seven days.

### Laboratory analysis

We ensured written consent was obtained before processing samples. Dry swabs were transferred into 10 ml of ThinPrep PreservCyt solution (Hologic Deutschland GmbH, Wiesbaden-Nordenstadt) and divided into 1 ml aliquots. The first-void urine, including pre-filled preservatives, was divided into 4 ml aliquots. HPV diagnostics were conducted using the Abbott m2000sp (Abbott GmbH & Co. KG, Wiesbaden) and Abbott m2000rt Real-Time PCR system (Abbott GmbH & Co. KG, Wiesbaden). Conducting a qualitative multiplex PCR assay to detect 14 high-risk HPV genotypes (16, 18, 31, 33, 35, 39, 45, 51, 52, 56, 58, 59, 66, and 68). It also distinguishes HPV-16 and -18 simultaneously and calculates the beta-globin cycle number (CN) to estimate DNA content. Signal intensity of beta-globin correlates with DNA amount and is represented by CN. This represents a cutoff when amplified DNA fragments during PCR are sufficient for analysis and is inversely proportional to the amount of DNA in the sample. All samples were processed as quickly as possible: urine samples within seven days and dry swabs within 30 days, as recommended by literature [22] [28]. Dry swabs were stored dry.

### Questionnaire

Patients were asked to complete two questionnaires: one to assess ease of use and satisfaction with the self-test device and the other for demographic information shown in the supplementary material (Table 1).

**Table 1:**
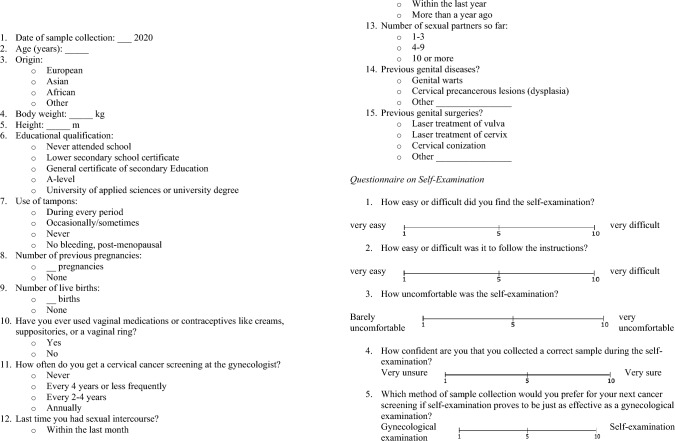
Questionnaires resulting in Demographics and user friendliness General Questionnaire

### Statistics

Participant rates were assessed using absolute and relative frequencies, odds ratio with 95% confidence interval, adjusted odds ratio, and a significance level of p < 0.05.

Sample quality was evaluated by analyzing absolute and relative frequencies of invalid samples. The CN of beta-globin was assessed using median, mean, standard deviation, minimum, maximum, lower 95% value, upper 95% value, ANOVA test, least significant difference (LSD) test, and visualized in a box plot.

HPV-results were presented using absolute and relative frequencies.

The satisfaction questionnaire consisted of non-validated scales and was analyzed descriptively (mean values, standard deviations, confidence intervals) and using ANOVA, and Chi-square tests.

Items from the general questionnaire were compared with the self-examination questionnaire using ANOVA, t-test, and Chi-square tests. Subgroup analyses by screening frequency were calculated using number of returned samples within each device group as the denominator.

A p-value < 0.05 was considered statistically significant. All analyses were descriptive unless statistical significance was demonstrated.

## Results

### Participation rates of the different self-tests

1860 samples of 19,995 distributed were returned, corresponding to 9.3%. 692 were opt-in (invited to request a self-test) and 1,168 opt-out samples (directly receiving a self-test); Only 16 samples were excluded due to transport damage or missing material; thus 1.844 samples were analyzed.

Opt-in: returns were highest for Colli-Pee (8.8%), second for FLOQSwabs (6.5%), and lowest for Evalyn (5.3%). Opt-out: returns were highest for FLOQSwabs (13.63%), second for Colli-Pee (12.04%), and lowest for Evalyn (9.39%). Colli-Pee and FLOQSwabs had higher return rates, without significance. The difference between opt-in and opt-out groups was statistically significant (p < 0.0001) for all tests (Table 2).

**Table 2:** Comparison of self-test kits by return rates, return rates by screening frequency (all percentages calculated using the number of returned samples within each device group as denominator), cycle numbers, HPV results, and user-friendliness outcomes (rating scale 1 (worst possible score) to 10 (best possible score)). Revealing a significantly higher return rate in the opt-out groups; increasing return rates for FLOQSwabs by decreasing screening frequency: lower CN mean for Evalyn brush; excellent sample quality; HPV results for each test kit; great user friendliness for all three self-testing tools.; preference for self-sampling

Self-test	Return rates		Return rates in different screening frequency groups		CN numbers		HPV results		Outcome Questionnaire						
										simplicity of examination	Simplicity of instructions	pleasant of examination	certainty of correct sample collection	favorite method	
olli-Pee	Opt-in	305/3455 (8.8%)	Missing	15 (38.5%)	Mean	24.37	positive	58 (8.31%)	Rating Mean	9.23	8.25	9.33	8.93	clinician taken sample	179 (27.4%)
	Opt-out	401/3330 (12.0%)	yearly	370 (36.7%)	SD	2.33	negative	635 (90.97%)	STD	1.41	2.31	1.40	1.69	self-testing	474 (72.6%)
	OR	0.707	every 2–3 years	199 (40.1%)	Min	19.47	invalid	5 (0.72%)	Missing (n)	27	25	29	26	Missing (n)	53
	95% CI; p-value	0.604; 0.828; p < 0.0001	every 4 + years	92 (38.8%)	Max	34.59			CI (95%)	8.36; 10.1	6.82; 9.68	8.46; 10.2	7.88; 9.98		
			never	30 (37.5%)											
Evalyn	Opt-in	178/3332 (5.3%)	Missing	12 (30.8%)	Mean	21.88	positive	42 (8.64%)	Rating Mean	9.02	9.16	8.73	8.10	Clinician taken sample	169 (37.6%)
	Opt-out	313/3334 (9.4%)	yearly	282 (28.0%)	SD	2.12	negative	440 (90.55%)	STD	1.84	1.53	2.03	2.22	self-testing	281 (62.4%)
	OR	0.545	every 2–3 years	131 (26.4%)	Min	17.64	invalid	4 (0.82%)	Missing (n)	12	12	13	12	Missing (n)	41
	95% CI; p-value	0.450; 0.659; p < 0.0001	every 4 + years	50 (21.1%)	Max	32.45			CI (95%)	7.88; 10.16	8.21; 10.11	7.47; 9.99	6.72; 9.48		
			never	16 (20.0%)											
FlOQSwabs	Opt-in	209/3213 (6.5%)	Missing	12 (30.8%)	Mean	24.09	positive	45 (6.82%)	Rating Mean	8.85	8.96	8.39	7.81	clinician taken sample	224 (36.4%)
	Opt-out	454/3331 (13.6%)	yearly	356 (35.3%)	SD	3.40	negative	604 (91.52%)	STD	1.87	1.71	2.27	2.24	self-testing	392 (63.6%)
	OR	0.441	every 2–3 years	166 (33.5%)	Min	17.93	invalid	11 (1.67%)	Missing (n)	21	15	19	18	Missing (n)	47
	95% CI; *p*-value	0.371; 0.523; *p* < 0.0001	every 4 + years	95 (40.1%)	Max	34.73			CI (95%)	7.69; 10.01	7.9; 10.02	6.98; 9.8	6.43; 9.2		
			never	34 (42.5%)											
Total	Opt-in	692/1860 (37.2%)							Rating Mean	9.04	8.74	8.83	8.31	clinician taken sample	572 (33.3%)
	Opt-out	1168/1860 (62.8%)							STD	1.71	1.96	1.96	2.1	self-testing	1147 (66.7%)
									Missing (n)	60	52	61	56	Missing (n)	141
	OR	0.5604							CI (95%)	7.98; 10.1	7.5; 9.95	7.62; 10.04	7.01; 9.61		
	95% CI; *p*-value	0.508; 0.618; *p* < 0.0001	*p*-value (CHI^2^)	0.1825	*p*-value (ANOVA)	*p* < 0.0001			*p*-value (ANOVA)	*p* = 0.0003	< 0.0001	< 0.0001	< 0.0001	*p*-Value (CHI^2^)	0.0003

Odds ratio, *CI* = confidence level, *n* = total number of participants answers/returned tests, *min* = minimum, *max* = maximum, *CN* = beta_globin cycle number, *HPV* = human papillomavirus; All numbers refer to the 1,860 ruterned samples of 19,995 distributed, analysed as per protocol population

Of note: Due to shipment error, 122 opt-in patients received Colli-Pee instead of FLOQSwabs, resulting in 2.5% more Colli-Pees than FLOQSwabs. Additional evaluation compared intention-to-treat (ITT) and as-tested populations. ITT ignores incorrectly sent kits; as-tested doesn´t. The resulting odds ratio remained unchanged. Thus, tables consistently assume the as-tested population to simplify analysis.

### Returns split by screening frequency and test

No significant difference was observed between screening frequency and return rates (Chi-square p = 0.1825). Return rates were calculated using number of returned samples within each device group as denominator. Among yearly screened participants, return rates were 28.0% for Evalyn, 36.7% for Colli-Pee, and 35.3% for FLOQSwabs. In women screened every 2–3 years, return rates were 26.4%, 40.1%, and 33.5%. For women screened every four years or less, return rates were 21.1%, 38.8%, and 40.1%. Among never-screened women, return rates were 20.0%, 37.5%, and 42.5%. FLOQSwabs showed a clear trend of increasing return rates with decreasing screening frequency. Additionally, returns for never-screened women were nearly twice as high for FLOQSwabs and Colli-Pee compared to Evalyn (Table 2).

### DNA collected

1844 samples were tested. The mean cycle number (collected DNA material) were: FLOQSwabs (24.09), Colli-Pee (24.36), and Evalyn (21.88). ANOVA and LSD test confirmed a significant difference between Evalyn and the other devices (p < 0.0001).

**Fig.1:**
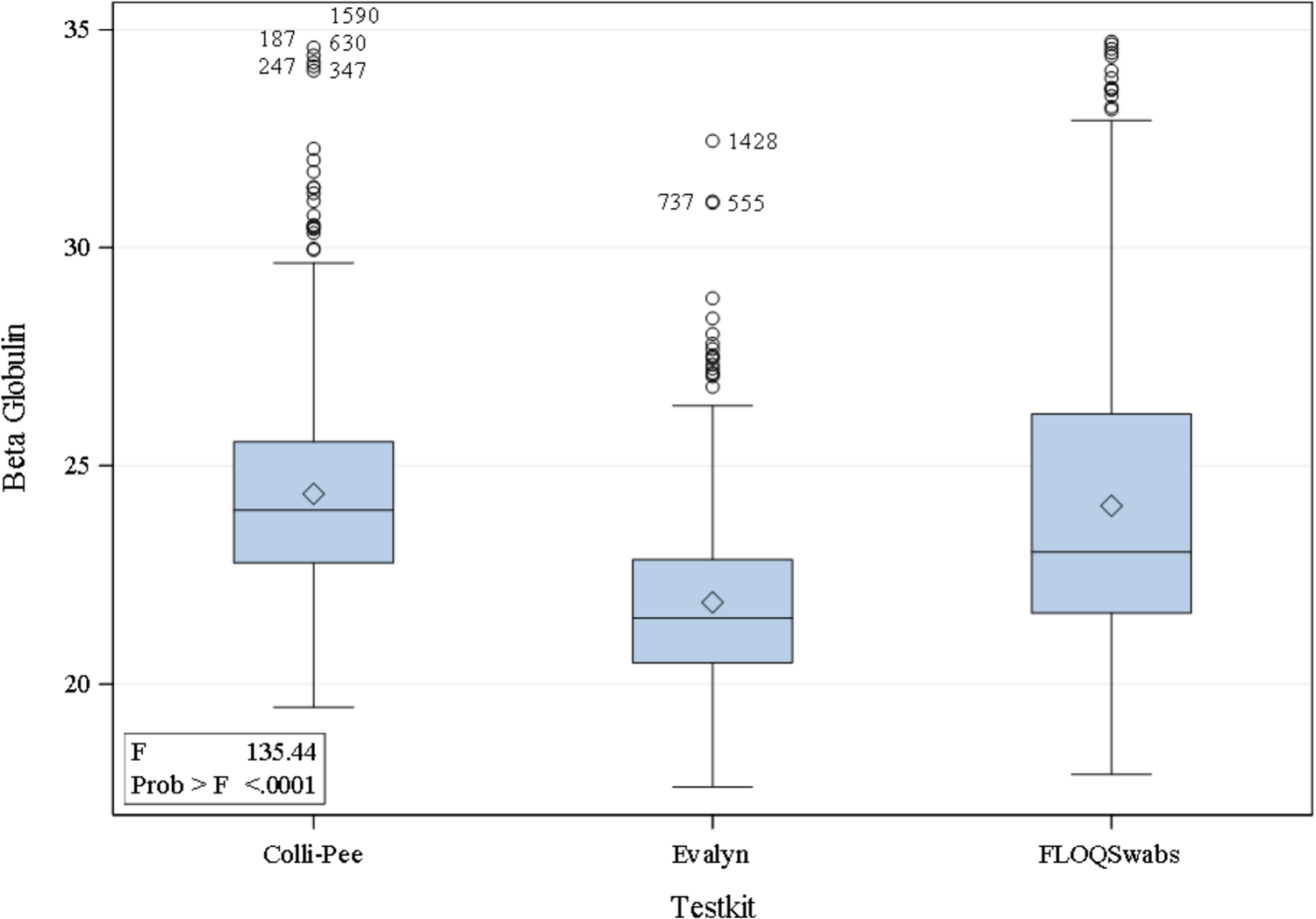
Beta globin cycle number by test kit, revealing a lower CN and therefore higher performance in DNA collection for Evalyn-Brush

The Boxplot (Fig. 1) shows, Evalyn had the highest DNA yield (lowest whisker), less variability (compact interquartile range), and consistent performance (tight clustering of data points around its median). FLOQSwabs and Colli-Pee showed no significant difference in mean values. However, Colli-Pee had the lowest ability to collect DNA material (highest lower whisker and highest outliers), while FLOQSwabs showed biggest variance (widest interquartile range and high outliers).

All devices performed excellently and collected sufficient DNA for HPV analysis. Only 20 of 1,844 samples were considered invalid due to insufficient cell material, resulting in 98.1% sample quality. FLOQSwabs invalidity was 1.67%; double compared to Colli-Pee (0.72%) and Evalyn (0.82%). Analysis confirmed high adequacy for HPV testing (Table 2).

### Human Papillomavirus

Evalyn had 42 (8.6%) positive results, Colli-Pee had 58 (8.3%), and FlOQSwabs had 45 (6.82%). FlOQSwabs had the highest number of invalid results due to insufficient cell content, as shown in Sect. "DNA collected" (Table 2).

### User-friendliness

Colli-Pee was significantly rated as easiest (mean 9.23) and most pleasant (mean 9.33). It also showed highest confidence in correct use (mean 8.93). For simplicity of instructions, Evalyn (mean 9.16) outperformed the other devices, but was rated slightly more complicated (mean 9.23), more unpleasant (mean 8.73), and less reliable (mean 8.10). FlOQSwabs ranked lowest in all categories except instruction simplicity, yet still scored very well (means 7.81—8.96).

Participants consistently preferred self-examination (66.7%) over gynecological examination (33.3%). Notably, Colli-Pee was most favored (72.6%), followed by FLOQSwabs (63.6%), and Evalyn (62.4%). Preference increased with decreasing screening frequency: yearly screened 57.1%, every 2—3 years 72.5%, every four years or less 85.3%, and never-screened 89.7%, showing a significant difference (p < 0.0001).

All self-tests were easy to understand and use, with high overall acceptability. Participants had a highly significant (p = 0.0003) preference for self-testing versus gynecological examination (Table 2).

### Demographics

1,600 participants were European, 60 from Middle East or Arab states, 26 from Africa, and 138 from another origin. Significant differences by origin were found for simplicity of examination (p < 0.0001) and simplicity of instructions (p = 0.0049). European participants rated both higher than African participants.

1,371 participants spoke German as their mother tongue, 433 another language. All material was provided in German only; German-speaking participants rated examination simplicity significantly higher (p < 0.0001). Interestingly, no significant difference was found for instruction simplicity (p = 0.5916). Thus, mother tongue was no hindrance in understanding provided instructions.

173 participants had a lower secondary school certificate, 579 a general certificate of secondary education, 329 an A-level, and 671 a university degree. Significant differences were observed for simplicity of examination (p < 0.0001) and simplicity of instructions (p = 0.0041).

From all evaluated user-friendliness parameters, only origin, mother tongue, and highest degree showed significant differences (Table 3).

**Table 3:** Influence of origin, mother tongue, and level of education on the questions: simplicity of examination and simplicity of instructions. Revealing an influence by origin and level of education on both questions, mother tongue only influenced simplicity of examination

	Origin of participants					Mother tounge			Level of education						Total
Simplicity of examination	missing (n)	Europe	Middle East, Arab states	Africa	other	missing (*n*)	german	other	missing (*n*)	No degree	Lower secondary school certificate	General certificate of secondary education	A-level	University of applied sciences or university degree	
n	36	1600	60	26	138	56	1371	433	41	67	173	579	329	671	1860
missing (n)	23	28	2	1	6	20	26	14	19	7	10	7	7	10	60
Rating mean/ median	8.15/ 10	9.12/ 10	8.17/ 9	8.04/ 9	8.76/ 9	8.69/ 10	9.15/ 10	8.70/ 10	7.95/ 10	7.98/ 9	8.83/ 10	9.11/ 10	9.12/ 10	9.12/ 10	9.04/ 10
STD	2.76	1.63	2.42	2.72	1.69	2.61	1.57	1.99	3.06	2.23	1.90	1.76	1.55	1.52	1.71
min/ max	02/10	01/10	01/10	01/10	02/10	01/10	01/10	01/10	01/10	01/10	01/10	01/10	01/10	01/10	01/10
p-value (ANOVA)	< 0.0001					< 0.0001			< 0.0001						

simplicity of instructions	missing (n)	Europe	Middle East, Arab states	Africa	other	missing (*n*)	german	other	missing (*n*)	No degree	Lower secondary school certificate	General certificate of secondary education	A-level	University of applied sciences or university degree	
n	36	1600	60	26	138	56	1371	433	41	67	173	579	329	671	1860
missing (n)	22	22	3	1	4	19	19	14	19	5	8	6	6	8	52
Rating mean/ median	8.71/ 10	8.76/ 10	8.56/ 9	7.40/ 9	8.87/ 10	8.62/ 10	8.73/ 10	8.79/ 10	8.36/ 9.5	8.13/ 8.5	8.66/ 10	8.96/ 10	8.75/ 10	8.64/ 9	8.74/ 10
STD	1.90	1.96	1.97	3	1.57	2.35	1.99	1.83	2.46	2.10	2.07	1.85	1.89	2.01	1.96
min/ max	04/10	01/10	01/10	01/10	04/10	01/10	01/10	01/10	01/10	01/10	01/10	01/10	01/10	01/10	01/10
p-value (ANOVA)	0.0049					0.5916			0.0041						

*n *= total number of answers, STD = standard deviation, the number Missing and Missing shows how many participants did not answer both questions

## Discussion

Our study's results demonstrate the potential effectiveness of implementing Evelyn, FlOQSwabs, or Colli-Pee in Germany´s regular screening program [29–31].

Screening frequency didn´t significantly affect return rates (Chi-square p = 0.1825), although Colli-Pee and FLOQSwabs showed slightly higher return rates than Evalyn. Subgroup analyses used returned samples by device as denominator. FLOQSwabs showed a clear trend of increasing return rates with decreasing screening frequency, whereas Evalyn had consistently lower return rates among underscreened women. This might suggest specific devices are more appealing to non-responders. Daponte et al. also report a preference for brush- or swab-based devices [3].

High sample quality is crucial for reliable HPV testing. Sample quality was excellent for all devices (98.1% validity), confirming reliability. FLOQSwabs had a marginally higher invalid rate, which may warrant further investigation. User feedback suggests FLOQSwabs may require more practice, as they were rated more challenging despite being rated simple in design and instructions. FLOQSwabs also showed highest variance in DNA collection, indicating inconsistent sampling. This may result from handling complexity or lower experience. Evalyn demonstrated highest efficiency in DNA collection, as indicated by its lower mean CN and more consistent performance, suggesting Evalyn might be most effective for DNA collection. Overall, all devices performed excellently without significant statistical differences.

These device-specific patterns were modest and should be interpreted cautiously, as most comparisons weren´t statistically significant and may reflect user characteristics rather than device performance. Overall, all three devices performed comparably in sample quality, HPV detection, and user-friendliness.

Integrating HPV self-testing into screening programs could increase participation, as seen in the Netherlands; participation increased from 7% (2017 + 2018) to 16% (2020) [32]. However, Dutch studies reported lower hr-HPV prevalence and follow-up rates for self-sampling [33, 34]; 99% of clinician samples had a cytological control versus 82% of self-tests, leaving 18% without follow-up [35]. Another Dutch study found only 5% missed follow-up after a doctor's visit, versus 14% among self-testers, potentially overlooking 21% of CIN 2 + lesions [36].

Validity of urine samples remains a topic of considerable debate. First-void urine may not always preserve DNA effectively, limiting nucleic acid extraction [37]. Compared to cervical samples, urine may collect cells from nearby areas, increasing HPV detection but lowering diagnostic accuracy [38–40]. Other studies show high correlation between first-void urine and vaginal samples, and urine self-sampling vs. clinician-collected samples [38, 39, 41].

Questionnaires showed high acceptance of all devices. Colli-Pee was rated easiest, most pleasant, and gave highest confidence in correct use. Evalyn outperformed in clarity of instructions, essential for proper use, and highlighting importance of clear design. Demographic analysis showed significant differences in ease of use and instruction clarity by origin, mother tongue, and education. Europeans, native German speakers, and those with higher education rated better, highlighting need for tailored communication.

A strong preference for self-testing was evident; 66.7% favored self-tests. Colli-Pee was most preferred (72.6%). Preference exceeded expectations from the preliminary study [28], indicating strong acceptance. Preference was significantly higher among underscreened women (89.7%) compared to yearly screened women (57.1%) (p < 0.0001). This could support early cancer detection. Our results align with former research [42, 43]. Reported advantages include absence of speculum examination, privacy, reduced embarrassment, comfort, social and cultural acceptability [26]. More recent studies reinforce this: A Spanish study (2020) revealed 87% preferred self-sampling. Significantly associated with younger age, higher education, and greater screening awareness [44]. An Italian study indicated preference increased with higher education and greater distance from regional screening centers [9]. Although our study found no influence on residency, this could be due to good infrastructure surrounding Hannover. Further research in other settings might yield different results.

Increasing participation in c.c. screening in Germany remains essential, as HPV vaccination uptake is lower than expected. Recent data from Barmer health insurance indicate 41% of girls and nearly 82% of boys haven´t been vaccinated against HPV [45].

A recent meta-analysis of 154 HPV self-sampling studies globally (482,271 women), found self-sampling nearly doubled screening likelihood (RR: 1.8; 95% CI: 1.7–2.0) [46].

Implementing one of the evaluated tests might lead to almost 80% of German women being screened and support reaching WHO´s goal of eliminating c.c..

Importantly, we otherwise undetected identified 145 h-HPV infections, 7.9% of 1860. Comparing HPV-results revealed: Evalyn had 8.6% positive results, Colli-Pee 8.3% and FlOQSwabs 6.82%. All devices demonstrated reliable HPV detection, reinforcing utility and effectiveness of self-sampling.

Limitations include voluntary participation, introducing selection bias and limiting generalizability. Subgroup analyses were descriptive and not powered to detect small device‑specific differences; findings should be interpreted cautiously. Finally, the study population consisted of women already engaged in screening, potentially underestimating challenges in reaching never‑screened individuals.

## Conclusion

Our results demonstrate high sample quality, reliable HPV detection, and high user acceptance of all three self-collection tests. Device-specific differences were small and did not reach statistical significance; they can therefore only be evaluated descriptively. Direct provision of user-friendly self-tests may support screening participation, particularly among underscreened women. Overall, all devices showed strong performance in DNA collection and appear suitable for integration into cervical cancer screening programs.

## Supplementary Information

Below is the link to the electronic supplementary material.

Supplementary file1 (DOCX 17 KB)

Supplementary file1 (PNG 366 KB)

## Data Availability

All data supporting the findings of this study are available within the paper and its Supplementary Information.
