# Supplementary material for: Comparison of three different hpv self-sampling tools – a subanalysis of the prospective, randomized hannover self-collection study
Source: Arch Gynecol Obstet. 2026 Jun 25;313(1):210. doi: 10.1007/s00404-026-08457-5 (PMC13303669; doi:10.1007/s00404-026-08457-5)
Supplement: Supplementary file 1 — Supplementary file1 (DOCX 17 KB) [file 404_2026_8457_MOESM1_ESM.docx]

**CONSORT 2010 Checklist – HaSCo Study Subanalysis**

**This checklist summarizes compliance with CONSORT 2010 reporting standards for the HaSCo subanalysis comparing self-sampling devices (Colli-Pee, Evalyn, FLOQSwabs).**

| **Section / Item** | **Description** | **Location in Manuscript** |
| --- | --- | --- |
| **Title and Abstract** | **Identifies study as a randomized subanalysis of the HaSCo trial; structured abstract includes objectives, design, participants, interventions, and outcomes.** | **Title page, Abstract** |
| **Introduction** | **Scientific background and rationale for comparing self-sampling devices; objectives clearly stated.** | **Introduction, paragraph 2** |
| **Methods** |  |  |
| **Trial design** | **Randomized, two-arm invitation design (opt-in vs. opt-out) with three self-sampling devices.** | **Methods 2.1** |
| **Participants** | **Women aged 30–65 years invited from population registry; inclusion/exclusion criteria described; women with annual screening participation excluded from main analysis.** | **Methods 2.1–2.2** |
| **Interventions** | **Description of Colli-Pee, Evalyn, and FLOQSwabs self-sampling devices; invitation procedures.** | **Methods 2.3** |
| **Outcomes** | **Primary outcome: sample quality and user-friendliness; secondary outcome: return rate and device performance.** | **Methods 2.4** |
| **Sample size** | **Based on total returned samples (n = 1,844); all included in subanalysis.** | **Methods 2.5** |
| **Randomization** | **1:1 allocation to opt-in vs. opt-out invitation; randomization method described.** | **Methods 2.1** |
| **Blinding** | **Not applicable (participants aware of invitation type).** | **Methods 2.1** |
| **Statistical methods** | **Descriptive and comparative analyses of sample quality and user experience; exclusion criteria applied post hoc for main study.** | **Methods 2.6** |
| **Results** |  |  |
| **Participant flow** | **Detailed in CONSORT flowchart (Figure 2); 19,995 invited, 1,860 returned, 16 excluded, 1,844 analyzed.** | **Results 3.1** |
| **Recruitment** | **Dates and process described; participation rates per device and invitation type reported.** | **Results 3.1** |
| **Baseline data** | **Demographics and screening history summarized in Table 2.** | **Results 3.2** |
| **Numbers analyzed** | **All returned samples (n = 1,844) analyzed; breakdown by device and annual screening participation provided.** | **Results 3.2** |
| **Outcomes and estimation** | **Comparative performance and user-friendliness results reported; confidence intervals provided.** | **Results 3.3** |
| **Ancillary analyses** | **Subanalysis of women with annual screening participation excluded from main HaSCo study.** | **Results 3.4** |
| **Harms** | **Not applicable (non-invasive self-sampling).** | **Results 3.5** |
| **Discussion** |  |  |
| **Limitations** | **Acknowledges inclusion of women with annual screening participation in subanalysis; discusses generalizability.** | **Discussion 4.1** |
| **Interpretation** | **Balanced interpretation of findings; comparison with previous studies.** | **Discussion 4.2** |
| **Generalizability** | **Applicability to population-based screening programs discussed.** | **Discussion 4.3** |
| **Other Information** |  |  |
| **Registration** | **DRKS registry number provided; protocol referenced.** | **Methods 2.1** |
| **Protocol** | **Protocol available upon request; deviations explained.** | **Supplementary Material** |
| **Funding** | **Supported by institutional and public health grants; no commercial influence.** | **Acknowledgments** |

**Note: The CONSORT checklist and flowchart are provided as supplementary material to ensure transparency and reproducibility of the HaSCo subanalysis.**
