# Supplementary figures and images for: Comparison of three different hpv self-sampling tools – a subanalysis of the prospective, randomized hannover self-collection study
Source: Arch Gynecol Obstet. 2026 Jun 25;313(1):210. doi: 10.1007/s00404-026-08457-5 (PMC13303669; doi:10.1007/s00404-026-08457-5)

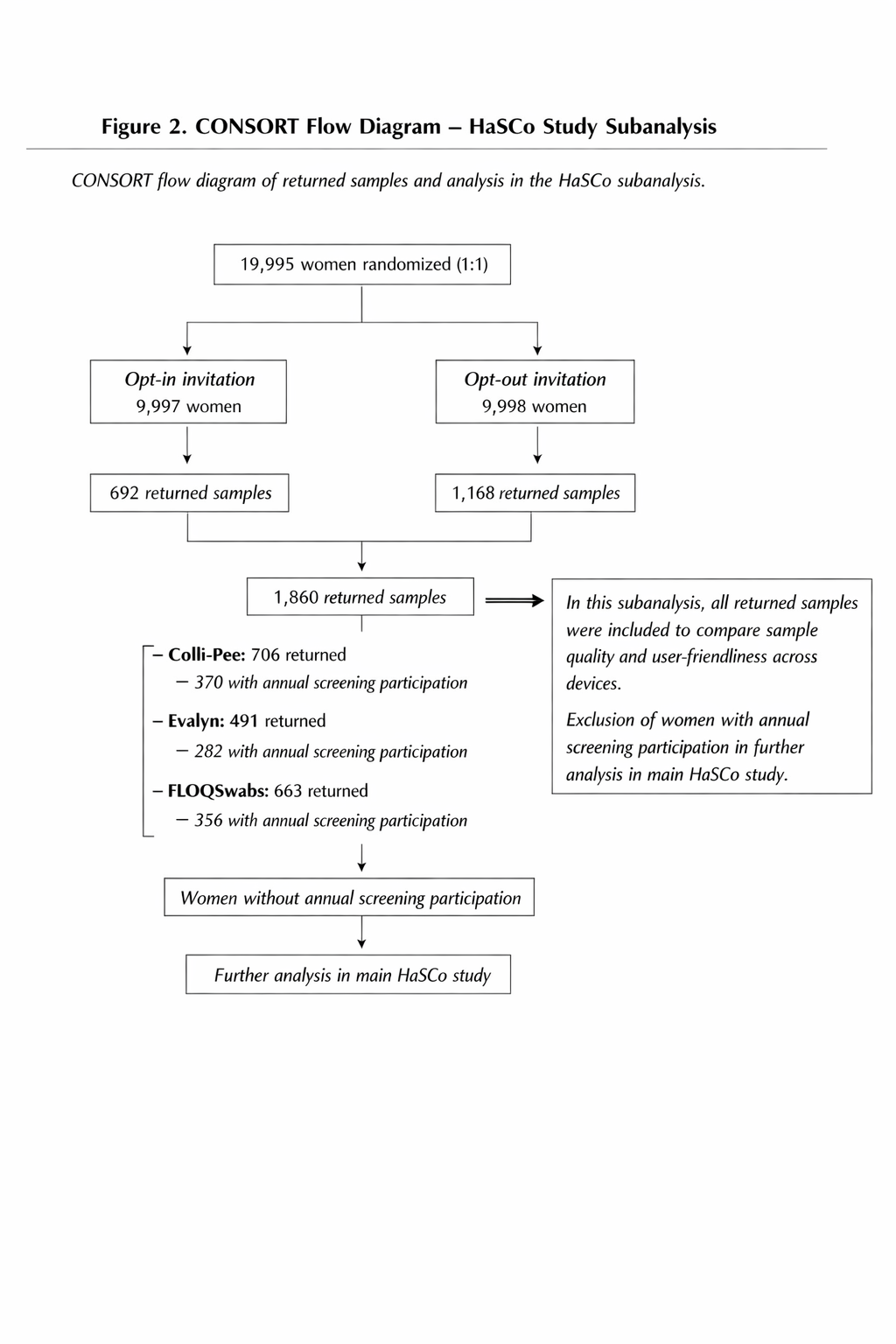

Supplement: Supplementary file 2 — Supplementary file1 (PNG 366 KB) [file 404_2026_8457_MOESM2_ESM.png]
